# Supplementary material for: SARS-CoV-2 Infection and C-Section: A Prospective Observational Study
Source: Viruses. 2021 Nov 22;13(11):2330. doi: 10.3390/v13112330 (PMC8622813; doi:10.3390/v13112330)
Supplement: Supplementary file 1 [file viruses-13-02330-s001.zip › viruses-1461653-suppl.pdf]

Supplementary Table S1. List of hospitals members of the Spanish Obstetric Emergency Group included in this study (n = 79).

**HOSPITAL**
AGSE Hospital Axarquia
Complejo Asistencial de León
Complejo Hospitalario A Coruña
Complejo Hospitalario de Jaén
Complejo Hospitalario San Millán y San Pedro
Complejo Hospitalario Universitario de Pontevedra
Complexo Hospitalario Universitario de Ourense
HM Hospital Nuevo Belén
Hospital Alto Guadalquivir
Hospital Álvaro Cunqueiro
Hospital Arnau de Vilanova
Hospital Clínico de Santiago de Compostela
Hospital Clínico San Carlos
Hospital Clínico San Cecilio (Complejo Hospitalario Universitario de Granada)
Hospital Clínico Universitario de Valladolid
Hospital Clínico Universitario Virgen de la Arrixaca
Hospital Costa del Sol
Hospital de la Santa Creu i Sant Pau
Hospital de Poniente
Hospital de Santa Caterina
Hospital de Son Llàtzer
Hospital de Torrejón
Hospital de Vinalopó
Hospital del Mar
Hospital del Tajo
Hospital d'Inca
Hospital do Barbanza
Hospital Doce de Octubre
Hospital Donostia
Hospital General de L'Hospitalet
Hospital General La Mancha Centro
Hospital General Universitario de Ciudad Real
Hospital General Universitario Gregorio Marañón
Hospital General Universitario Santa Lucía
Hospital Infanta Margarita
Hospital Jerez de la Frontera
Hospital La Fe
Hospital La Línea
Hospital Parc Taulí
Hospital Puerta de Hierro
Hospital Quirón Pozuelo de Alarcón
Hospital Quirónsalud Málaga
Hospital Rafael Méndez

Hospital Regional Universitario de Málaga
Hospital Reina Sofía
Hospital San Pedro Alcántara
Hospital Sant Joan de Reus
Hospital Santa Ana
Hospital Universitari Dexeus - Grupo Quirónsalud
Hospital Universitari Germans Trias i Pujol
Hospital Universitario Araba-Txagorritxu
Hospital Universitario Central de Asturias
Hospital Universitario de Basurto
Hospital Universitario de Burgos
Hospital Universitario de Cabueñes
Hospital Universitario de Ceuta
Hospital Universitario de Ferrol
Hospital Universitario de Fuenlabrada
Hospital Universitario de Girona Doctor Josep Trueta
Hospital Universitario de Salamanca
Hospital Universitario de Tarragona Juan XXIII
Hospital Universitario de Torrevieja
Hospital Universitario Doctor Peset
Hospital Universitario Infanta Sofía
Hospital Universitario Juan Ramón Jiménez
Hospital Universitario La Paz
Hospital Universitario Puerta del Mar
Hospital Universitario Río Hortega
Hospital Universitario Son Espases
Hospital Universitario Torrecárdenas
Hospital Universitario Virgen de las Nieves
Hospital Universitario Virgen de Valme
Hospital Universitario Virgen del Rocío
Hospital Universitario Virgen Macarena
Hospital Universitario de Getafe
Hospital Universitario Severo Ochoa
Hospital Viamed Santa Ángela de la Cruz
Hospital Virgen de la Concha
Hospital Virgen de la Luz

Supplementary Table S2. STROBE Statement—checklist of items that should be included in reports of observational studies

	Item No.	Recommendation	Page No.	Relevant text from manuscript
**Title and abstract**	1	(*a*) Indicate the study's design with a commonly used term in the title or the abstract	1	
		(*b*) Provide in the abstract an informative and balanced summary of what was done and what was found	2	
**Introduction**				
Background/rationale	2	Explain the scientific background and rationale for the investigation being reported	2-3	
Objectives	3	State specific objectives, including any prespecified hypotheses	3	
**Methods**				
Study design	4	Present key elements of study design early in the paper	3-4	
Setting	5	Describe the setting, locations, and relevant dates, including periods of recruitment, exposure, follow-up, and data collection	3-4, Figure 1 and Supplementary Table S1	
Participants	6	(*a*) *Cohort study*—Give the eligibility criteria, and the sources and methods of selection of participants. Describe methods of follow-up<br><br>*Case-control study*—Give the eligibility criteria, and the sources and methods of case ascertainment and control selection. Give the rationale for the choice of cases and controls<br><br>*Cross-sectional study*—Give the eligibility criteria, and the	3-4 and Figure 1	

		sources and methods of selection of participants	
		(*b*) *Cohort study*—For matched studies, give matching criteria and number of exposed and unexposed<br>*Case-control study*—For matched studies, give matching criteria and the number of controls per case	
Variables	7	Clearly define all outcomes, exposures, predictors, potential confounders, and effect modifiers. Give diagnostic criteria, if applicable	3-4 and registry protocol: https://osf.io/xspwq/<br>https://osf.io/m5yps/
Data sources/ measurement	8*	For each variable of interest, give sources of data and details of methods of assessment (measurement). Describe comparability of assessment methods if there is more than one group	3-4 and registry protocol: https://osf.io/xspwq/<br>https://osf.io/m5yps/
Bias	9	Describe any efforts to address potential sources of bias	4 and 13
Study size	10	Explain how the study size was arrived at	3-4 and Figure 1
